# 4-Aminopyridine Does Not Enhance Flocculus Function in *Tottering*, a Mouse Model of Vestibulocerebellar Dysfunction and Ataxia

**DOI:** 10.1371/journal.pone.0057895

**Published:** 2013-02-25

**Authors:** John S. Stahl, Zachary C. Thumser

**Affiliations:** 1 Neurology Division, Louis Stokes Cleveland Dept. of Veterans Affairs Medical Center, Cleveland, Ohio, United States of America; 2 Department of Neurology, Case Western Reserve University, Cleveland, Ohio, United States of America; University of Iowa, United States of America

## Abstract

The potassium channel antagonist 4-aminopyridine (4-AP) improves a variety of motor abnormalities associated with disorders of the cerebellum. The most rigorous quantitative data relate to 4-AP's ability to improve eye movement deficits in humans referable to dysfunction of the cerebellar flocculus. Largely based on work in the ataxic mouse mutant *tottering* (which carries a mutation of the Cacna1a gene of the P/Q voltage-activated calcium channel), 4-AP is hypothesized to function by enhancing excitability or rhythmicity of floccular Purkinje cells. We tested this hypothesis by determining whether systemic or intrafloccular administration of 4-AP would ameliorate the eye movement deficits in *tottering* that are attributable to flocculus dysfunction, including the reductions in amplitude of the yaw-axis vestibulo-ocular reflex (VOR) and vision-enhanced vestibulo-ocular reflex (VVOR), and the optokinetic reflex (OKR) about yaw and roll axes. Because *tottering's* deficits increase with age, both young and elderly mutants were tested to detect any age-dependent 4-AP effects. 4-AP failed to improve VOR, VVOR, and OKR gains during sinusoidal stimuli, although it may have reduced the tendency of the mutants' responses to VOR and VVOR to decline over the course of a one-hour recording session. For constant-velocity optokinetic stimuli, 4-AP generated some enhancement of yaw OKR and upward-directed roll OKR, but the effects were also seen in normal C57BL/6 controls, and thus do not represent a specific reversal of the electrophysiological consequences of the *tottering* mutation. Data support a possible extra-floccular locus for the effects of 4-AP on habituation and roll OKR. Unilateral intrafloccular 4-AP injections did not affect ocular motility, except to generate mild eye elevations, consistent with reduced floccular output. Because 4-AP did not produce the effects expected if it normalized outputs of floccular Purkinje cells, there is a need for further studies to elucidate the drug's mechanism of action on cerebellar motor dysfunction.

## Introduction

4-aminopyridine (4-AP) and the related pyridine derivative 3,4-diaminopyridine (3,4-diAP) are non-selective antagonists of voltage-activated potassium channels, including those underlying the classical A-type K^+^ current, the K_v_1-mediated D-type current, and some delayed rectifier-currents [1], [2]. Long used experimentally to pharmacologically dissect the contributors to net ionic currents of neurons, the aminopyridines have also found several medical applications, including restoring the function of the neuromuscular junction in the paraneoplastic condition Lambert-Eaton myasthenic syndrome and improving action potential conduction through axons whose myelin has been damaged by multiple sclerosis [3]–[5]. More recently, the aminopyridines have been used to treat abnormal motor function caused by disorders of the cerebellum, with particular emphasis on the eye movement abnormalities attributable to dysfunction of the vestibulocerebellum [6]. 4-AP and/or 3,4-diAP have been variously demonstrated to attenuate vertical instabilities of the eyes (both downbeat and upbeat nystagmus), enhance smooth pursuit eye velocity and the gain of the vertical VOR, and to lengthen the abnormally short time constants of the horizontal and vertical neural integrators [7]–[11]. Patients who experienced benefit have suffered cerebellar dysfunction stemming from a variety of etiologies, with and without radiologically demonstrable cerebellar atrophy [7], [9]. Aminopyridines have also been demonstrated to prevent ataxic episodes in patients with episodic ataxia type 2 (EA-2), a calcium channelopathy caused by a broad range of mutations of the CACNA1A gene of the P/Q calcium channel [12]. In *tottering*, an ataxic mouse carrying a mutation in the homologous Cacna1a gene, aminopyridines prevent episodes of stress-induced dystonia [13] and improve performance on the accelerating rotarod test [14].

The impact of aminopyridines on cerebellar dysfunction in humans and mice has been ascribed most often to an enhancement of Purkinje cell excitability or synaptic transmission between Purkinje cells and their synaptic targets (for instance, see [7], [13], [15], [16]). More recently, an alternative theory has been advanced in which aminopyridines alter the rhythmicity of Purkinje cell firing [14]; Purkinje cell firing has been demonstrated to be highly irregular in *tottering*, the mutant's loss of normal cerebellar function has been ascribed to the irregularity [17], [18], and 4-AP was shown to restore regularity of *tottering* Purkinje cells in the slice preparation [14]. The lattermost study also provided evidence against 4-AP having any effect on mean Purkinje cell firing rate, or the response of Purkinje cells to parallel fiber inputs, or the efficiency of Purkinje cell synaptic contacts with their targets. Note that while discarding the idea that 4-AP increases Purkinje excitability or synaptic efficacy, the new concept of 4-AP function (which has already entered the clinical literature, see [12]) still has 4-AP working on Purkinje cells so as to enhance/normalize the influence of cerebellar cortex on its synaptic targets.

While the idea that aminopyridines somehow normalize Purkinje cell signals has experimental support, it also depends on some extrapolations of uncertain validity. The idea that 4-AP increases Purkinje cell excitability was derived from its effects over a limited concentration range on calcium spiking in slices prepared from normal guinea pigs [19], [20]. The relevance of those findings to sodium (simple) spike activity in Purkinje cells affected by P/Q channel mutations and other cerebellar disorders in intact animals is unknown. The idea that the benefits of 4-AP devolve from changes in rhythmicity likewise rests on its effects in the slice preparation [14]. The location of the recordings was not reported, but likely did not include the flocculus (since that lobule is difficult to preserve in the mouse slice preparation), even though the flocculus is the region of the cerebellum for which there is the strongest basis to hypothesize connections between changes in signal content/transmission and behavioral improvements following administration of aminopyridines. Caution is required when extrapolating from experiments outside the vestibulocerebellum to the vestibulocerebellum (i.e., the nodulus, ventral uvula, and flocculus), since the complement of potassium channels on Purkinje cells exhibits regional variability and vestibulocerebellar Purkinje cells may be particularly specialized [21]. Thus the degree to which 4-AP regularizes firing in floccular Purkinje cells in the slice is unproven. More important, it is unknown whether 4-AP regularizes Purkinje cell firing at any site in intact animals, where the inputs to the cerebellum (which are destroyed in the slice preparation) could potentially introduce influences on Purkinje cell rhythmicity that override the aminopyridine effect seen in the slice.

Even if 4-AP does regularize firing in floccular Purkinje cells of intact calcium channelopathy mice, it should be recognized that the role of irregular firing in cerebellar dysfunction is itself speculative. The idea was first advanced in an *in vivo* study of floccular Purkinje cells in *tottering*, because rhythmicity was the only assayed Purkinje cell property that was found to be abnormal [17]. Since many of *tottering*'s ocular motor deficits could be mimicked by flocculectomy, the authors argued that the irregularity was in some manner nullifying the transmission of otherwise normal Purkinje cell signals to their brainstem targets. However, it remains possible that other properties that Hoebeek et al. did not assay are actually responsible for the dysfunction, and dysrhythmicity is merely an epiphenomenon. In particular, no assessment has ever been performed of population-based properties (e.g., synchronicity or numbers of modulating cells) of *tottering* floccular Purkinje cells. Subsequent studies supporting the dysrhythmicity theory [14], [18] are even more limited in this respect; whereas the Hoebeek et al. study did assess the normality of firing rate modulation with respect to eye movements, the subsequent studies focused on regions of the cerebellum in which the normal modulation patterns of single Purkinje cells are unknown. Moreover, most of the experiments were conducted in the slice preparation. Consequently the possibility that the mutation alters modulation patterns or communication of that modulation to synaptic targets could not be explored. The potential for dysrhythmicity to degrade the transfer of modulation from Purkinje cells to their synaptic targets has been supported by modeling [22] but also disputed [23]; the principle has yet to be demonstrated experimentally in the flocculus-vestibular nucleus system. Finally, Purkinje cell irregularity has been demonstrated primarily in mice whose ataxia originates from alterations of calcium and calcium-dependent currents [17], [18], [24]. The aminopyridines have been demonstrated to improve vestibulocerebellar dysfunction (principally, but not exclusively, downbeat nystagmus) stemming from a broad range of etiologies [7], [9]. The vast majority of the documented patients were not known to suffer a channelopathy, and it is unknown whether any of their disorders are accompanied by disordered Purkinje cell rhythmicity. If not, then their responsiveness to 4-AP indicates the existence of mechanisms of action other than increasing Purkinje cell regularity, and if such alternative mechanisms exist, then they may be the mechanisms that are actually responsible for improving behavioral performance in the mouse mutants that do exhibit Purkinje cell irregularity and increased regularity under the influence of aminopyridines.

It is important from the standpoint of medical uses of aminopyridines to resolve uncertainties in the mechanism by which they ameliorate cerebellar dysfunction [14], [25]. Because of the non-specificity of their action and the wide distribution of sensitive potassium channels within the central nervous system, aminopyridines have an exceedingly narrow therapeutic range and a high propensity to cause adverse effects, including nausea, paresthesias, lightheadedness, dizziness, nervousness, and most significantly, seizures, at serum levels not far above the minimum effective level [26]–[28]. It would be desirable to identify an agent with greater specificity, one capable of reproducing the aminopyridine effect but also better tolerated. More specific agents could be screened for their ability to regularize Purkinje cell activity in mutants like *tottering*, but this approach will ultimately fail if Purkinje cell dysrhythmicity is not actually the cause of the motor deficits responding to aminopyridines.

Studying the effects of aminopyridines on eye movements of ataxic mice such as *tottering* can provide insight into the drug's mechanism of action. A multiplicity of neuroanatomical and electrophysiological studies in mice and other mammals lacking a fovea/area centralis has produced a general understanding of the signals emanating from the cerebellar flocculus, their brainstem destinations, and their immediate influences on compensatory eye movements, i.e., the eye movements made reflexively in response to rotation of the animal or its visual surround [17], [29]–[37]. In afoveate mammals, Purkinje cells modulate their simple spike firing rate during compensatory eye movements driven either by rotation of the animal (i.e., during the vestibulo-ocular reflex, VOR) or during rotation of the visual surround about the animal (optokinetic reflex, OKR). Although Purkinje cells are inhibitory, the sense of their modulation is such that the output of the flocculus generally augments the eye movement response. Thus if 4-AP normalizes flocculus modulation or the efficacy of its transmission to brainstem targets, it should enhance the gains of the VOR and OKR. It also follows from the neuroanatomy and effects of electrical stimulation [33], [38] that if 4-AP increases floccular Purkinje cell mean firing rate (or again, the transmission of the mean firing rate to floccular targets in the vestibular nuclei) *in vivo*, then drug treatment should cause each eye to rotate approximately about the preferred axes of the ipsilateral anterior and horizontal semicircular canals, producing a downward and lateral shift of mean eye position. Because such predictions are possible with regard to the cerebellar flocculus and eye movements, and not possible for other regions of the cerebellum participating in control of limb or axial musculature, the flocculus/eye movement system is the best setting in which to investigate the mechanism of aminopyridine actions on cerebellum-related motor deficits.

There is a wide variety of ataxic mouse strains in which the effects could be tested, but there are numerous reasons to focus on *tottering*. These include: 1) The eye movements of *tottering* (as well as the allelic Cacna1a mutant, *rocker*) have been investigated extensively [39], [40]; 2) Eye movement deficits in *tottering* accord well with predicted effects of flocculus hypofunction and exhibit strong parallels to eye movement deficits of humans with vestibulocerebellar dysfunction (see more, below); 3) *Tottering* cerebellum exhibits relatively little cell loss, at least when young [41]. Thus motor dysfunction can be attributed to abnormal signaling rather than absence of neurons. It seems particularly plausible that a drug could correct the signaling abnormality, whereas compensation for loss of cellular elements may be more problematic. Along these lines, differential responsiveness of mutants to 4-AP prior to and after developing Purkinje cell loss has been demonstrated in a mouse model of spinocerebellar ataxia type 1 [42]. 4) In humans, some aspects of ocular motor dysfunction have been argued to be more responsive to aminopyridines when they are found in the context of radiologically demonstrable cerebellar atrophy [9]. Elderly *tottering* do develop progressive atrophy [41], [43], so testing this strain at young and old age provides the opportunity to examine the effects of aminopyridines in the context of varying degrees of cerebellar atrophy; 5) As reviewed above, many of the studies that form the basis for speculations regarding the actions of aminopyridines were performed in *tottering*.

The parallels between the eye movement deficits in *tottering* and humans with cerebellar dysfunction deserve emphasis. Humans with disorders affecting the cerebellar flocculus exhibit an array of ocular motor abnormalities, including impaired smooth pursuit and a related impairment of the ability to suppress the VOR when tracking a target moving with the head, impaired ability to maintain eccentric gaze (corresponding to reductions in the time constant of the brainstem ‘neural integrator’), impaired VOR plasticity, post-saccadic drift, and downbeat nystagmus (DBN) [44]. All of these deficits can be replicated in non-human primates by specific lesions of the flocculus [45]. *Tottering* likewise exhibits reduced time constants of the neural integrator and VOR plasticity [39], [40]. Mice, like other afoveate mammals, lack smooth pursuit of visual targets and other aspects of the ocular motor repertoire related to foveal vision. However, their floccular Purkinje cells do support the eye movement response to rotation of the entire visual surround. Purkinje cells of afoveate mammals responding to vertical motion of the surround increase their simple spike firing rate during downward motion, which corresponds nicely to the finding that primate floccular Purkinje cells underlying smooth pursuit increase firing during downward tracking, and the observation that the human flocculus increases its metabolic activity during downward tracking [46], [47]. Furthermore, lesions of the flocculus in mice recapitulate *tottering*'s OKR deficit [17], just as lesions in primates recapitulate the smooth pursuit deficits. To date, DBN has not been described in ataxic mice, but *tottering* exhibits hyperactivity of the ocular response to static pitch tilts [48], which may parallel the hyperactivity of the otolith circuitry that has been proposed to contribute to the tilt-dependent component of DBN in humans [49], [50].

We assessed the effect of subcutaneous administration of 4-AP on eye movements in *tottering*, concentrating on eye movement abnormalities that had been demonstrated in the previous behavioral study [40], were relatively robust, and could be assayed quickly enough to allow a before- and after-drug assessment of the behavior in a single experimental session. Although 4-AP did engender subtle changes in eye movements, it did not produce the effects that would be expected if it normalized the function of floccular Purkinje cells, indicating a need for further studies to elucidate the drug's mechanism of action on cerebellar motor dysfunction. Preliminary results have been reported [25], [51].

## Methods

### Animals and animal preparation

Animal experiments were approved by the Institutional Animal Care and Use Committees at the Louis Stokes Cleveland Dept. of Veterans Affairs Medical Center (protocol 08-111-MS-003) and Case Western Reserve University (protocol 2009-0024), and followed the recommendations in the Guide for the Care and Use of Laboratory Animals of the National Institutes of Health. All surgery was performed under isoflurane anesthesia, and all efforts were made to minimize suffering.

C57BL/6 control animals were obtained from The Jackson Laboratory (Bar Harbor, ME) and *tottering* mutants were bred locally as previously described [40]. Both sexes were used. Mutants were divided into young (2–8 months) and old cohorts (14–22 months). As reviewed in Introduction, the use of an old cohort provided an opportunity to assess effects of aminopyridines that could appear only in the context of cerebellar atrophy. C57BL/6 mice ranged 4–8 months of age. Prior work has demonstrated that ocular motility in C57BL/6 remains stable at least through 18 months of age [39], so a single broadly aged group of C57BL/6 provides an adequate control for both *tottering* age groups. For intrafloccular injection experiments, mutants were aged 3–8 months.

Animals were prepared for recording by surgical implantation of an acrylic head-fixation pedestal as previously described [52]. During implantation the animals were held in a stereotactic frame so that the top of the completed pedestal accurately paralleled the lambda-bregma axis. For the animals undergoing intrafloccular injection of 4-AP, a craniotomy was fashioned in the interparietal bone and surrounded by a built-up acrylic chamber joined to the fixation pedestal.

### Recording apparatus

We assessed the effects of 4-AP on compensatory eye movements elicited by vestibular stimulation about the yaw axis in darkness (vestibulo-ocular reflex, VOR) and light (vision-enhanced vestibulo-ocular reflex, VVOR) and by optokinetic stimulation (optokinetic reflex, OKR) about the yaw and roll axes. Vestibular stimuli were generated by a servo-controlled turntable and optokinetic stimuli by a planetarium projector. The animal was surrounded by a cylindrical, light-tight curtain system that allowed testing in darkness and also provided a uniform surface for projection of the planetarium's ‘star’ pattern. While testing VVOR, the planetarium was illuminated and held stationary, thereby providing a high-contrast stationary visual surround. During recording the animal's head pedestal was bolted to a support armature and its body was loosely restrained in an acrylic tube. The support armature was angled so that when placed in the recording apparatus, the lambda-bregma axis was pitched 20° nose-down.

Eye movement recording was accomplished using a commercial pupil-tracking video oculography system (ETL-200, ISCAN, Inc., Burlington, MA), operating under infrared illumination at a sampling rate of 120 Hz. The video oculography camera could be yawed ±10° about a vertical axis passing through the recorded eye to provide calibration data [39], [53], [54]. The recorded eye was treated with 0.5% physostigmine salicylate to limit pupil dilation in the dark. The horizontal and vertical positions of the pupil, horizontal and vertical positions of a reference corneal reflection, and pupil diameter were output from the oculography system as analog signals. These signals, along with turntable position and planetarium velocity, were passed through four-pole Bessel low-pass filters (corner frequency 200 Hz) and then stored to a digital acquisition system (System 3, Tucker-Davis Technologies, Gainesville, FL) at a rate of 500 samples/s. Raw eye position signals were converted by the trigonometric procedures detailed previously [39], [53] to horizontal angle with respect to each animal's average resting position, and vertical elevation angle with respect to the earth-horizontal plane. Note that because the 4-AP treatment had the potential to alter vertical position of the eye, the calculation of horizontal eye position was adjusted to take into account the vertical position of the eye [39].

### Natural stimuli

We concentrated on facets of compensatory eye movements that had previously been demonstrated to be most consistently abnormal in *tottering* and an allelic Cacna1a mutant, *rocker*, and are also consistent with decrements in the normal influences of the cerebellar flocculus [17], [39], [40]. Among the qualifying abnormal behaviors, we selected specifically those amenable to rapid assessment, allowing for pre- and post-treatment data sets to be collected in a single recording session. Furthermore, we designed the stimulus battery to minimize the duration of each recording session, since compensatory eye movements in mice may decline during long sessions, and Cacna1a mutants appear particularly prone to this ‘habituation’ [39]. Each session included determination of resting position during stationary conditions in the light, 0.8 Hz ±4.8° sinusoidal VVOR, 0.8 Hz ±4.8° VOR, constant-velocity yaw and roll OKR over a ±2.5–40°/s range, and sinusoidal 0.4 Hz ±4° yaw and roll OKR. Each sinusoidal record was 40 seconds in duration. As in our previous studies, the constant-velocity OKR stimuli consisted of 4-second, constant-velocity periods of alternating direction separated by 3.5-s periods of darkness during which the planetarium reversed direction and reaccelerated to the next test speed. The constant-velocity and sinusoidal OKR provide overlapping but complementary assessments of optokinetic function. The constant-velocity stimulus generates a direct assessment of a critical non-linear property of the OKR, its strong dependence on stimulus velocity. However, the magnitude of the response to constant-velocity stimuli depends partly on the animal generating fast phases of nystagmus. The fast phases involve neural apparatus unrelated to the optokinetic pathways, and in mice they can fail after the first few seconds of exposure to the constant-velocity stimulus [55]. The sinusoidal stimulus, with its continuously changing velocities, is more difficult to interpret with respect to the speed tuning of the optokinetic system, but the low-amplitude stimulus triggers relatively few fast phases, and therefore is more purely dependent on the function of optokinetic pathways. In the interest of speeding the data collection, we saved time re-orienting the planetarium by collecting all the yaw-axis responses, followed by the roll-axis responses.

### Drug preparation and conduct of systemic injection experiments

During each session, animals received a subcutaneous injection of either 1.25 mg/kg 4-AP or normal saline, in both cases at a volume of 4.2 ml/kg. 4-AP (#275875, Sigma-Aldrich) was diluted in normal saline at a concentration 3 mg/10 ml and titrated to a neutral pH (6.8–7.2) with HCl. Our 4-AP dose was slightly greater than the minimum dose of 4-AP that was effective in preventing dystonic attacks in *tottering* [13]. (Weisz *et al*. did not test higher dosages of this agent, and in our own pilot experiments, we found that higher dosages often triggered squinting behavior under our head-fixed conditions, a behavior suggesting non-tolerance and one that was incompatible with recording eye movements.) Additional data from an earlier set of drug and saline experiments at a lower 4-AP dosage (mean±SD dosage 0.73±0.13 mg/kg, range 0.48–0.97) were available and were explored where they could provide insight into repeatability of findings at the higher dosage. (The presence of a qualitatively similar drug effect at the lower dosage would support there having been a true drug effect in the higher-dosage experiments, whereas the absence of the effect would be non-informative, as the lack of the effect could simply be attributable to the dose being insufficient.)

Prior to the recording session, a fine-gauge hypodermic needle connected to an infusion pump (PHD2000, Harvard Apparatus) containing the injectate was inserted subcutaneously in the loose skin of the dorsal neck. All air was purged from the injection system prior to needle insertion. The animal was then positioned in the rotation apparatus and calibration data acquired. Pre-treatment yaw-axis responses were collected, cycling through the stimulus battery until we had collected a minimum of three records of each sinusoidal stimulus and four repetitions of the constant-velocity stimulus. Then the planetarium axis was reoriented and the roll-axis sinusoidal and constant-velocity responses were collected, similarly alternating the stimuli. Next, the injection was performed, followed by a 10-minute period to allow for drug absorption, followed by collection of post-treatment responses (first roll, then yaw). The post-treatment data collection was completed in an average of 19 minutes, well within the expected duration of action of the drug [56]. The entire duration of the data collection period (including pre-treatment, absorption, and post-treatment phases) averaged 56 minutes. Saline injection and 4-AP injection experiments were conducted in identical fashion and alternated, with no more than one session conducted per day. Except where noted, each animal contributed two drug sessions and two saline sessions to the results of the high-dose 4-AP experiments.

### Drug preparation and conduct of intrafloccular injection experiments

In animals whose rotational responses were to be studied before and after intrafloccular injection of 4-AP, the animal was prepared with a craniotomy and recording chamber as described above. Several preliminary recording sessions were conducted to identify the coordinates of the approximate center of the left flocculus. The flocculus was identified by the presence of Purkinje cells whose complex spikes were modulated in response to rotatory optokinetic stimuli about either the vertical (yaw) axis, or about a horizontal axis deviated 135° from the nose toward the left side [17]. The subsequent data acquisition sessions were conducted using a double-barreled micropipette with a recording channel connected to the extracellular amplifier, and a drug channel coupled to a pressure injection unit (Picospritzer II, Parker Hannifin, Pine Brook, NJ). The recording channel was filled with 2 M NaCl and 4% Fast Green, and the drug channel was filled with a 500 µM solution of 4-AP in an artificial CSF solution composed of 126 mM NaCl, 26 mM NaHCO_3_, 3 mM KCl, 1.2 mM KH_2_PO_4_, 1.6 mM MgSO_4_, 5 mM HEPES and titrated to pH 7.4. Dextrose and calcium were omitted from the artificial CSF vehicle after preliminary experiments suggested that these components may have increased the tendency for the injection channel to plug. The 4-AP concentration was selected with the following considerations: 1) The IC_50_ of 4-AP on the Purkinje cell A-current (which has been cited as the mechanism of the drug's therapeutic effect [16]) is 68 µM [57]; 2) The IC_50_ of 4AP on the voltage-dependent potassium current in Purkinje cell dendrites is 100 µM [58], coinciding with the concentration commonly used in *in vitro* studies in which the 4-AP is bath-applied; 3) If a total ejectate volume of 20 nL (the midpoint of our target delivery for the 10-minute induction period) were allowed to expand to fill a 400 nL space (a rough approximation of the volume of the mouse flocculus) of which only 21% is accessible interstitial space [59], its concentration would fall to about 20%. In fact, the concentration likely falls considerably further, since ejectate is not confined to the flocculus, being subject to loss up and down the electrode track, clearance by the circulation, and diffusion to more distant cerebellum; 4) Since pilot experiments did not suggest a clear effect of intrafloccular 4-AP injections, we elected a relatively high concentration, to reduce the possibility that a lack of effect in the final results would be attributable to insufficient dosage.

Preliminary experiments indicated the injection volumes (which are estimated at the conclusion of the experiment, see below) would vary over a considerable range. Even if the drug's beneficial effects were limited to a specific concentration range and that range was surpassed in some experiments, the effective concentration should still be achieved in experiments in which smaller volumes were delivered, and the drug effect should be detectable in plots of drug effect vs. injection volume. It was expected (and proved) that injections in some experiments would be trivial (only a few picoliters of injectate delivered over the course of the induction period). These unsuccessful sessions served as control (sham) injection sessions.

The animal was mounted in the rotation apparatus and the electrode was advanced into a Purkinje cell layer in the approximate center of the left flocculus, after which a set of pre-treatment responses to the rotation battery (first yaw, then roll) was acquired. Next, single 100 ms duration pressure pulses were applied to the drug channel while monitoring the neural recording. The success of the ejection was demonstrated by an immediate attenuation of all neural signals. If ejection failed (presumably due to plugging of the electrode tip), longer ejections at higher pressures were attempted. Once an effective ejection was achieved, ejection was repeated every 30 seconds for a 10-minute induction period, after which post-treatment rotation responses (first roll, then yaw) were collected. We continued to inject every 30 seconds at the same injection parameters (pressure, pulse duration) throughout the collection of the post-treatment data.

Immediately after post-treatment data were gathered (and without further advances in electrode position, which might alter ejection characteristics due to plugging or tip breakage), the electrode was removed and placed in a bath of paraffin oil under a microscope. Ejections were made into the oil using the same pressure and duration parameters, the spheres of ejectate were photographed, their diameters measured, and ejectate volume/injection was calculated.

### Data analysis

Eye movement responses to sinusoidal rotational stimuli were analyzed as previously described [53]. Briefly, periods of the record with interruptions (e.g. due to loss of the video signal, chewing/struggling artifacts, or failure to construct a clean slow phase signal due to excessive numbers of fast phases) or non-alertness were deleted. In the remaining cycles, fast phase eye movements were detected and patched by linear interpolation from the surrounding slow phase data. The patched cycles were then averaged and Fourier analysis was performed on the stimulus and eye velocity records to extract the amplitude at the stimulus frequency. Gain was calculated as the ratio of response to stimulus amplitudes.

Additional analyses were performed on sinusoidal OKR data to determine if 4-AP generated differential effects on gain during upward and downward eye movements (a possibility raised by the post-4-AP responses to constant-velocity stimuli). For this hemicycle analysis, we began by processing the data to remove signal dropouts and saccades and to generate the averaged response. Second, planetarium velocity was fit with a sinusoid and the eye and planetarium records were each divided into two halves based on where the fitted planetarium velocity was positive (i.e., upward with respect to the recorded eye) or negative. Third, multiple linear regression was performed on the upward and downward hemicycles for the eye and planetarium using the model equation Y(t) = A sin 2πft+B cos 2πft+C, where A, B, and C are regression coefficients, f is the stimulus frequency, and t is time. Fourth, the amplitudes of each hemicycle were calculated according to the equation |Y| = √(A^2^+B^2^). Fifth and finally, the hemicycle gains were calculated by the ratio of the amplitudes (|Y|) of the eye velocity and planetarium velocity.

Eye movement responses to constant-velocity OKR were analyzed as previously described [39]. For each period of constant-velocity planetarium rotation, periods of signal loss were deleted, fast phases and their immediate aftermath were ‘patched’ by linear interpolation from the flanking constant-velocity slow phase movements, average velocity was calculated, and gain was calculated as the ratio of the eye and planetarium velocities. Note that as described previously in mice [55], on occasion animals would cease making resetting fast phases, causing the eye to arrest in an eccentric position. Such behavioral arrests were excluded from the calculation of gain. We also excluded the first few tens of milliseconds after a fast phase when the eye position often exhibits a rapidly declining exponential profile, rather than the linear profile typical of the response to constant-velocity OKR. These portions likely reflect post-saccadic glissades [60] and as such are driven by pulse-step mismatches (i.e., mismatches between the eye position dictated by the pulse of saccadic innervation and that specified by the corresponding step of output from the neural integrator) rather than the optokinetic circuitry. Plots of gain versus stimulus frequency (speed-tuning curves) were then compiled.

To calculate resting eye position, we first excluded sections of each record where eye tracking was lost, then selected all portions of the record in which the eye was stable and calculated from them the average eye position. On occasion animals executed a saccade, and after briefly holding the new position, would return to the original region, either via an exponential drifting movement or via a saccade. Such transient eccentric positions were excluded. In cases in which there were linear drifts without an identifiable stable equilibrium position, the entire drifting record was used. Likewise, on occasions where there were repeated saccades and it was unclear which stable eye positions represented the equilibrium region, we averaged all of the stable eye positions.

Throughout, treatment-related changes in measures are calculated as post-treatment minus pre-treatment values. Average values are reported as mean±SD except where noted. All statistical significance levels are based on unpaired *t*-tests except as noted. All statistical tests are 2-tailed.

## Results

### Systemic 4-AP effects on rotation responses

The effects of systemic injections of 4-AP 1.25 mg/kg or saline on rotation responses were determined in 12 sessions in 6 young *tottering*, 10 sessions in 5 old *tottering*, and 10 sessions in 5 C57BL/6 control animals (in all cases, 2 drug sessions and 2 saline sessions for each animal). Data from the earlier experiments at lower 4-AP dosage included 15 4-AP and 10 saline sessions in 5 young *tottering*, 17 4-AP and 13 saline sessions in 8 old *tottering*, and 15 4-AP and 10 saline sessions in 5 C57BL/6 controls. Note that every animal contributed data to both the saline and drug databases.

**Figure 1:**
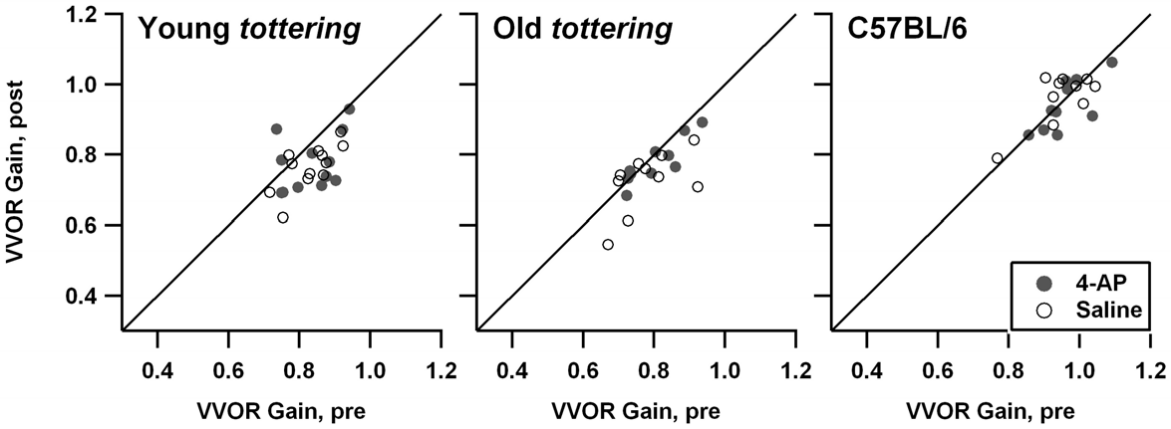
Comparison of visually enhanced vestibulo-ocular reflex (VVOR) gain before and after saline (open symbols) or 1.25 mg/kg 4-AP. Each animal cohort is represented in a separate plot. Diagonal line indicates a 1∶1 post-treatment:pre-treatment relationship. Mutants exhibited some decline in gain from pre-treatment to post-treatment, suggesting habituation. There was no effect of 4-AP.

**Figure 2:**
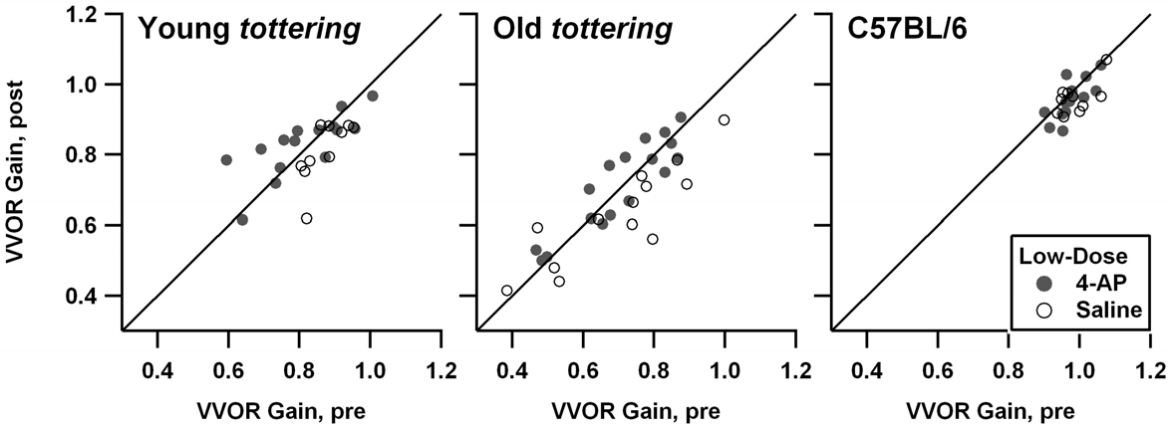
Comparison of vestibulo-ocular reflex (VVOR) gain before and after saline or 4-AP, in the low-dose experimental series. Symbols and format are identical to Figure 1. This larger series of experiments with a somewhat differing set of animals suggests that 4-AP can reduce habituation in the mutants.

In a prior behavioral study, *tottering* exhibited decreased gains during rotation in the light (vision-enhanced vestibulo-ocular reflex, VVOR) as compared to control animals, particularly around 0.8 Hz [40]. Figure 1 compares 0.8 Hz VVOR gain before and after treatment. Each cohort (young *tottering*, old *tottering*, and controls) is shown in its own subplot, with filled symbols representing 4-AP sessions and open symbols representing saline sessions. Any points falling on the diagonal line would indicate a session in which pre- and post-treatment gains were identical. Comparison of the *tottering* and control subplots indicates that, as in the prior study, the *tottering* cohorts exhibited lower average VVOR gain as compared to the control group. Both old and young *tottering* tended to have lower gains in the post-treatment period (most data points fall below the diagonal line marking a 1∶1 post:pre-treatment relationship), indicating a tendency for animals to habituate (or fatigue) over the course of the recording session. Control animals did not exhibit this decline. A similar contrast in the degree of habituation for mutants and controls was observed in a behavioral study of the allelic Cacna1a mutant *rocker* [39], raising the possibility that a greater tendency to habituate is a hallmark of loss-of-function Cacna1a mutations, not specific to the exact mutation. Filled and open symbols in Figure 1 overlap perfectly, indicating that 4-AP had no effect on *tottering's* subnormal VVOR gain. On the other hand, the larger series of low-dose 4-AP (which was performed with a partially non-overlapping set of animals) raises the possibility of an effect of 4-AP on habituation in the mutants. Figure 2 shows the scatterplots from that series. For young *tottering*, the post-treatment minus pre-treatment changes in VVOR gains averaged -0.060±0.060 for the saline sessions vs. +0.019±0.076 for the 4-AP sessions, which differ significantly (p = 0.011). For old *tottering*, the 4AP effect was likewise significant, the gain change averaging −0.069±0.089 for saline vs. +0.009±0.058 for 4-AP (p = 0.007).

**Figure 3:**
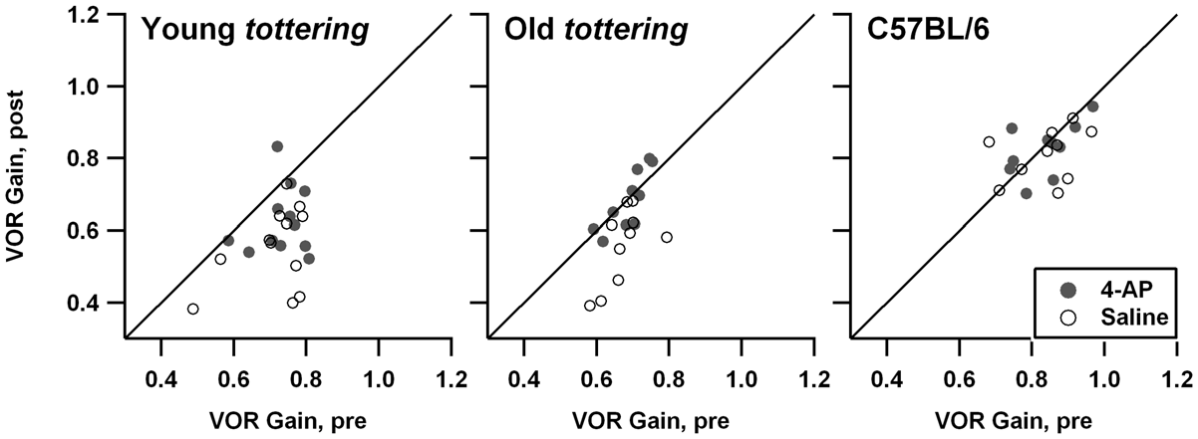
Comparison of vestibulo-ocular reflex (VOR) gain before and after saline or 1.25 mg/kg 4-AP. Symbols and format are identical to Figure 1. 4-AP may have reduced habituation in the elderly *tottering* cohort.

Figure 3 shows the analogous figures for the response to 0.8 Hz VOR. The results are similar to the VVOR results. Once again, there was a suggestion of a reduction in habituation following 4-AP, although it was restricted to the old *tottering* cohort. The difference of pre- and post-treatment VOR gains for old *tottering* was −0.114±0.082 for the saline sessions vs. −0.004±0.050 for the 4-AP sessions, a significant difference (p = 0.0019). The possibility of a true 4-AP effect is re-enforced by the data from the low-dose 4-AP series, in which the habituation was reduced by 4-AP, although in that case the significant difference appeared in the young mutant cohort. In that series, the differences of pre- and post-treatment VOR gains for young *tottering* averaged −0.100±0.082 for the saline sessions vs. −0.021±0.072 for the 4-AP sessions (p = 0.0093), and for old *tottering*, −0.066±0.145 for saline vs. −0.042±0.095 for 4-AP (p = 0.59). The lack of a statistical effect for the old animals is owed in large part to a single saline session in which the pre-treatment gain was unusually low (0.044) by comparison to the animal's other sessions, resulting in a large increase post-saline (to 0.362), as the animal reverted toward its more customary gain. Omitting this session, the average post-treatment minus pre-treatment change for the old *tottering* saline sessions was −0.098±0.092, which brings the SD more in line with those of other cohorts/conditions, and improves the p-value for the 4-AP vs. saline *t*-test to p = 0.13. Thus even at the lower dosage of 4-AP, there was a drug-associated trend toward less VOR habituation.

**Figure 4:**
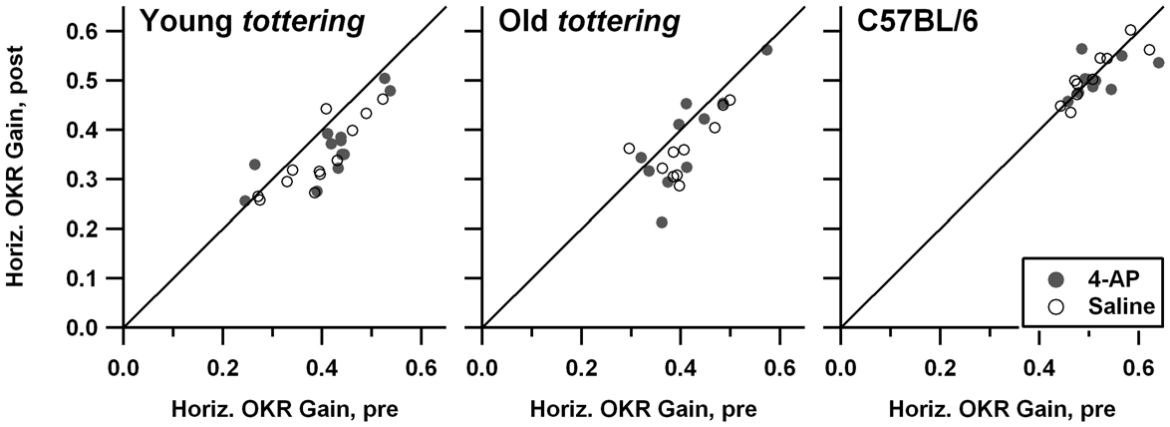
Comparison of yaw sinusoidal optokinetic reflex (OKR) gain before and after saline or 1.25 mg/kg 4-AP administration. Symbols and format are identical to Figure 1. Mutants exhibited habituation, without evidence of a 4-AP effect.

**Figure 5:**
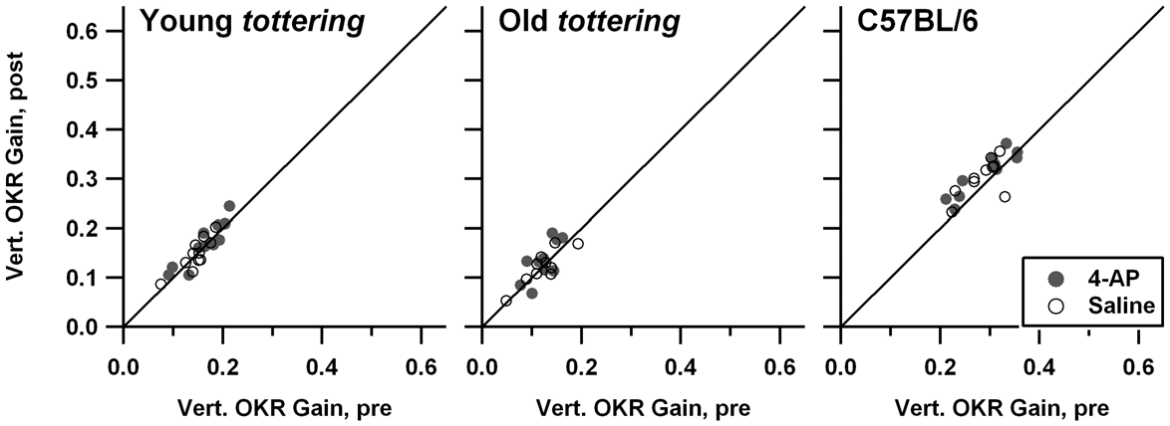
Comparison of roll OKR gain before and after saline or 1.25 mg/kg 4-AP administration. Symbols and format are identical to Figure 1. There was no evidence of a 4-AP effect. In contrast to yaw OKR, overt habituation did not occur.

Figure 4 shows the pre/post-treatment comparisons for sinusoidal OKR in yaw. Comparison of the *tottering* and C57BL/6 subplots indicates that as in the prior behavioral study [40], *tottering* exhibited lower optokinetic gains in the yaw plane. The current set of 5 old and 6 young mutants did not replicate the earlier observation, based on larger cohorts, that roll OKR gain declines as *tottering* ages, but that age-related decline *was* replicated in the larger cohorts of the lower-dose 4-AP experiments. As was the case for VVOR and VOR, *tottering* exhibited a tendency for gain to be lower in the post-treatment period, as more points fall below the 1∶1 line than above. There was no effect of the drug on yaw OKR gains in the mutants. Inspection of the comparable plots from the low-dose 4-AP series (plots not shown) do not suggest either habituation or any effect of 4-AP on gain. Figure 5 shows the analogous figures for roll OKR. *Tottering* was previously shown to have markedly lower gains for constant-velocity roll OKR [40], and Figure 5 indicates that the deficit is also present for a sinusoidal stimulus; the *tottering* data points are clearly shifted leftward and downward as compared to the data from C57BL/6. Saline and 4-AP data points overlap perfectly, indicating the lack of any drug effect. It is interesting that while there was no evidence of habituation in the mutants (data points are distributed symmetrically about the 1∶1 line), control animals exhibited a slight *increase* in gain post-treatment. The low-dose 4-AP experiments (plots not shown) were essentially identical, exhibiting a lack of habituation in the mutants, and an ‘anti-habituation’ in the controls, without any drug effect on gain in any cohort. Note that one cannot exclude the possibility that the mutants' lack of the control animals' post-treatment increment relates to the habituation exhibited by mutants during VOR, VVOR, and yaw OKR; habituation could be perfectly offsetting what would otherwise be a post-treatment gain increase.

**Figure 6:**
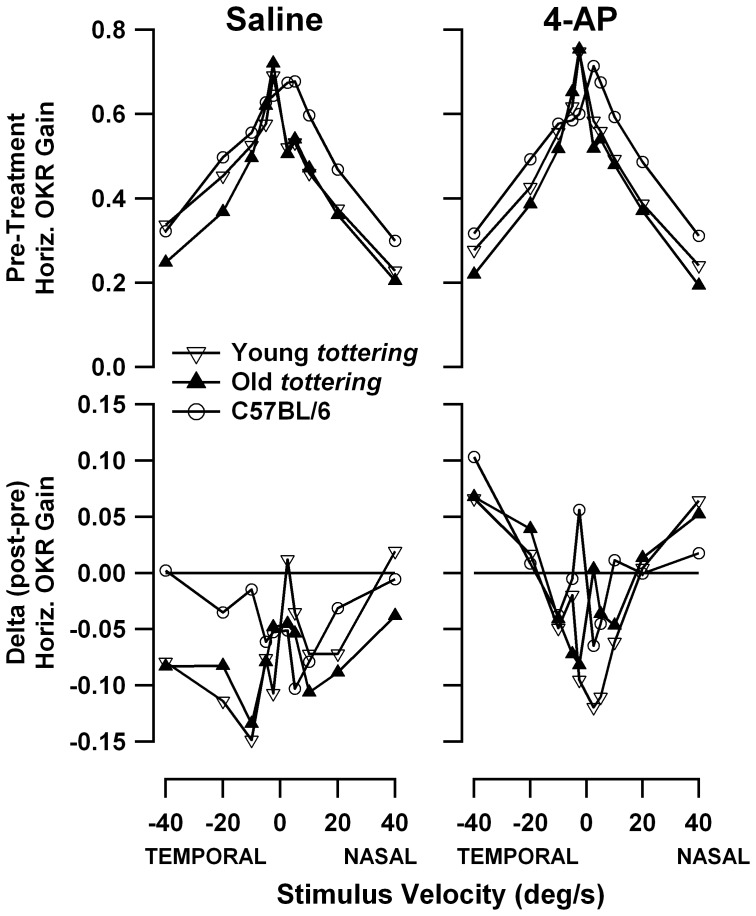
Speed tuning curves for yaw OKR. Top panels depict the average speed tuning curves in the pre-treatment period, with one curve for each animal cohort. Bottom curves plot the difference between average gain curves before and after treatment (post-treatment minus pre-treatment). A horizontal line indicating no change is superimposed for reference. Left panels are for saline sessions and right panels are for 4-AP sessions. All cohorts exhibited habituation, with mutants probably more affected than controls. 4-AP may have reduced the habituation mildly.

Figure 6 compares the speed tuning curves for constant-velocity yaw OKR before and after treatment. The top panels show the complete tuning curves in the pre-treatment period and the bottom panels show the point-wise differences between the post-treatment and pre-treatment speed tuning curves. Saline sessions are shown in the left panels, and 4-AP sessions on the right. As in the prior behavioral study of *tottering* [40], *tottering* yaw OKR gain was reduced compared to controls. As in the results for the sinusoidal yaw OKR described above, young and old mutants exhibited similar deficits, but in the larger cohorts of the lower-dose 4-AP experiments series (plots not shown), older animals exhibited the more severely depressed gains reported in the earlier study. In the saline experiments, gains generally declined slightly post-treatment for mutants and controls (difference curves lie mostly below the reference line indicating zero gain change). The results following 4-AP are complex, but suggest lesser post-treatment declines for all three cohorts at the higher stimulus velocities. Averaging across all velocities, the gain changes averaged −0.030±0.058 for 4-AP and −0.067±0.044 for saline in young *tottering*, −0.010±0.059 for 4-AP and −0.076±0.042 for saline in old *tottering*, and +0.004±0.038 for 4-AP and −0.043±0.036 for saline in controls. The 4-AP vs. saline comparison was statistically significant for old *tottering* (p = 0.010) and controls (p = 0.010) but missed significance for young *tottering* (p = 0.094). Thus there may be a tiny effect of 4-AP on the gain of constant-velocity yaw OKR, but the effect size (measured by the difference in gain changes between 4-AP and saline sessions) is very small and may not represent a specific reversal of the neural signaling abnormalities underlying the mutant's behavioral deficits, since the effect was observed in the control animals as well. If it is a real effect (as opposed to a random one), then it could be another manifestation of the potential effect of 4-AP on habituation that was described with the VOR data, above. However, the fact that 4-AP did not influence habituation during sinusoidal yaw OKR argues against the existence of a generalized anti-habituation effect.

**Figure 7:**
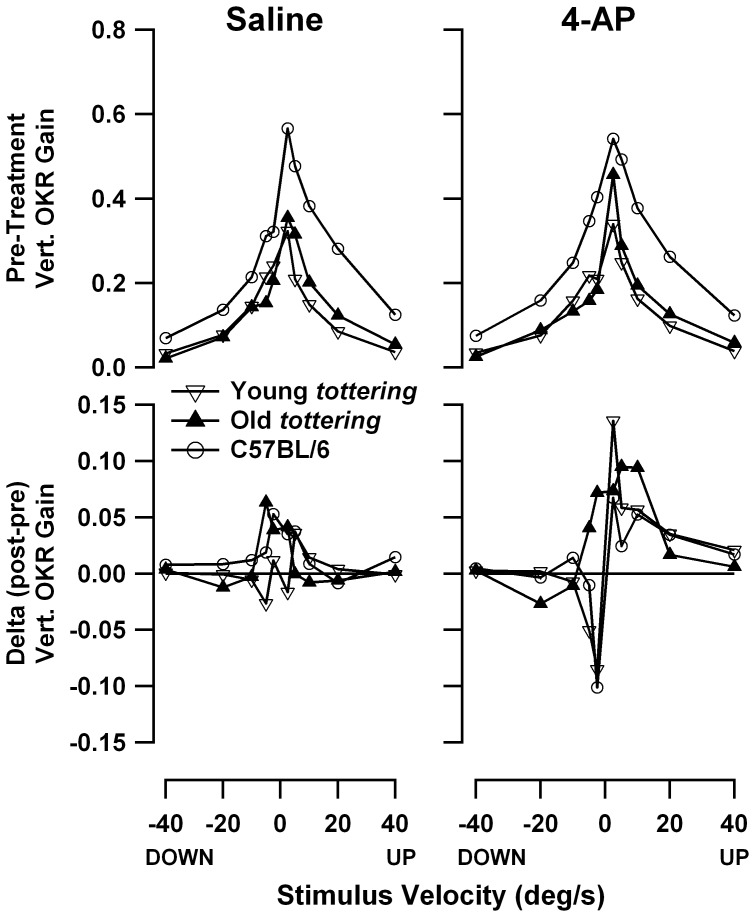
Speed tuning curves for roll OKR. Format is identical to Figure 6. 4-AP enhanced gains during upward stimulation for all cohorts. In contrast to the yaw OKR, habituation is not in evidence.

**Figure 8:**
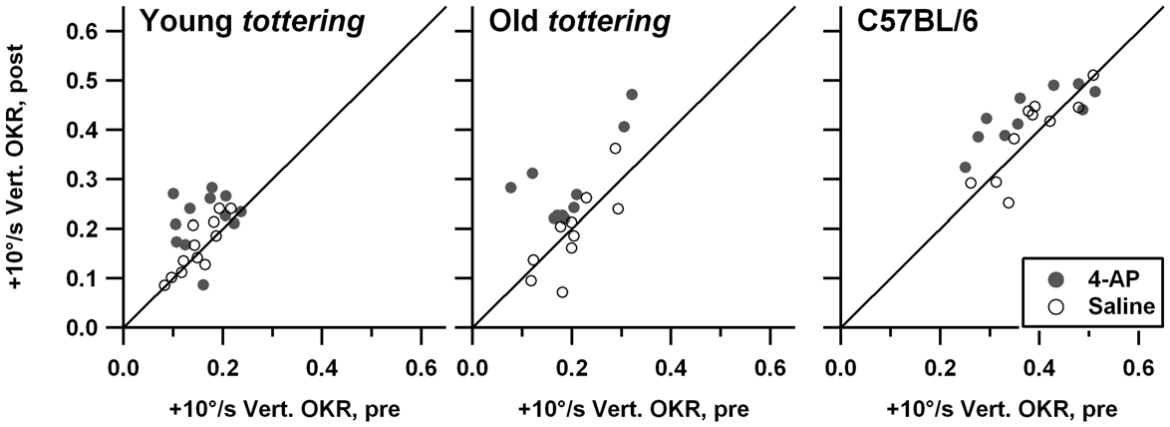
Comparison of response to +10°/s constant-velocity roll optokinetic stimulation before and after saline or 4-AP, in the format of Figure 1. These data generated the +10°/s points in the averaged speed tuning data of Figure 7. The scatterplots indicate that the 4-AP effect seen in the average tuning curves derived from multiple sessions, and not from a very few outliers.

Figure 7 compares the speed tuning curves for constant-velocity roll OKR, with lower panels showing, as in Figure 6, the post-treatment minus pre-treatment gain differences. As for the constant-velocity yaw data described above, the mutants exhibited the gain deficiencies seen in the prior study [40], excepting the tendency for older animals to have more severe deficits. However once again, the age effect *was* observed in the larger series of experiments performed at lower 4-AP dosages (plot not shown). There was essentially no change in gain following administration of saline. Controls and old *tottering* exhibit some elevation above the zero-change reference line, but this is of uncertain significance, as it is limited to the lowest stimulus speeds, where tiny variations in eye velocity can cause relatively large swings in the gain calculation. In contrast, following 4-AP administration, gain changed for all 3 cohorts. In the elderly mutants, gain was broadly increased within the ±10°/s range. For young mutants and controls, gain was increased for upward movements and decreased for low-speed downward movements. The changes in young mutants and controls are fairly symmetrical at the low speeds, but the increase in upward gains extends to higher speeds than is the case for the decrease in downward gains. Figure 8 shows the post- vs. pre-treatment scatterplots for +10°/s, the median upward speed tested and a point at which the pre-treatment tuning curves of mutants and controls differ strongly in this and the prior behavioral study. The figure demonstrates that the increased post-treatment gains in the averaged tuning curves are representative of the trend across all data, rather than the effect of a few outlier sessions. Paired *t*-tests indicate that post-4-AP gain differed significantly from pre-4-AP gain for all cohorts (young *tottering* p = 0.012; old *tottering* p = 0.0014; control p = 0.020), while pre/post-saline values did not (young *tottering* p = 0.10, old *tottering* p = 0.64, control p = 0.56). Plots analogous to those in Figure 8 (not shown) compiled from the low-dose 4-AP experiments show a similar elevation of upward gains after 4-AP in the mutants that was not observed in saline sessions, although for the control animals, the upward gains are elevated post-treatment in the saline as well as the 4-AP sessions.

The gain changes in roll-axis OKR following 4-AP could originate from several sources. One possibility is that the response to upward-directed image velocity is actually increased. If so, the gain change should also be detectable in the response to our 0.4 Hz sinusoidal stimuli (provided that the putative enhancement in upward gain isn't restricted to stimulus frequencies below 0.4 Hz). We analyzed 0.4 Hz OKR gains separately during the upward and downward hemicycles of the planetarium motion, as described in Methods. Inspection of the post- vs. pre-treatment scatterplots (not shown) indicate no tendency to greater upward hemicycle gains following 4-AP. The post-treatment minus pre-treatment differences in upward hemicycle gain in the 4-AP sessions averaged +0.016±0.057 for young *tottering* (paired *t-*test p = 0.35), −0.003±0.11 for old *tottering* (p = 0.94), and +0.021±0.027 for controls (p = 0.039). While there was a marginally significant increase for controls, that group also exhibited an increase following saline. Therefore the difference does not explain the increase in upward gains during constant-velocity roll that was specific to 4-AP sessions.

4-AP could also have caused some of the changes in gain seen in Figure 7 if it had engendered an upward drift of the eyes, on which an otherwise unchanged optokinetic response was superimposed. This possibility is suggested by the observation that gain changes in young *tottering* and controls exhibited some symmetry around speed zero, with gain increases for positive speeds and decreases for negative speeds. It can be demonstrated that adding an upward drift of the eye of just 0.2°/s to the optokinetic response would generate the apparent increase in gain of 0.1 at +2.5°/s. (The calculation is based on the pre-treatment gain of 0.34 for young *tottering* responding to +2.5°/s optokinetic stimulation.) We assessed whether an upward bias appeared during roll OKR by calculating mean eye velocities during the sinusoidal roll OKR and determining the post-drug minus pre-drug eye velocity differences. By cohort, the saline vs. 4-AP velocity differences were −0.14±0.20°/s vs. −0.14±0.17°/s for control, +0.06±0.28 vs. −0.14±0.53 for young *tottering*, and +0.29±0.27 vs. +0.09±0.46 for old *tottering*. Thus if anything, 4-AP produced (in mutants only) a weak *reduction* (i.e., less positivity or greater negativity) of mean eye velocity as compared to saline, making it unlikely that the post-4-AP gain changes in Figure 7 are attributable to the superposition of an upward velocity bias.

**Figure 9:**
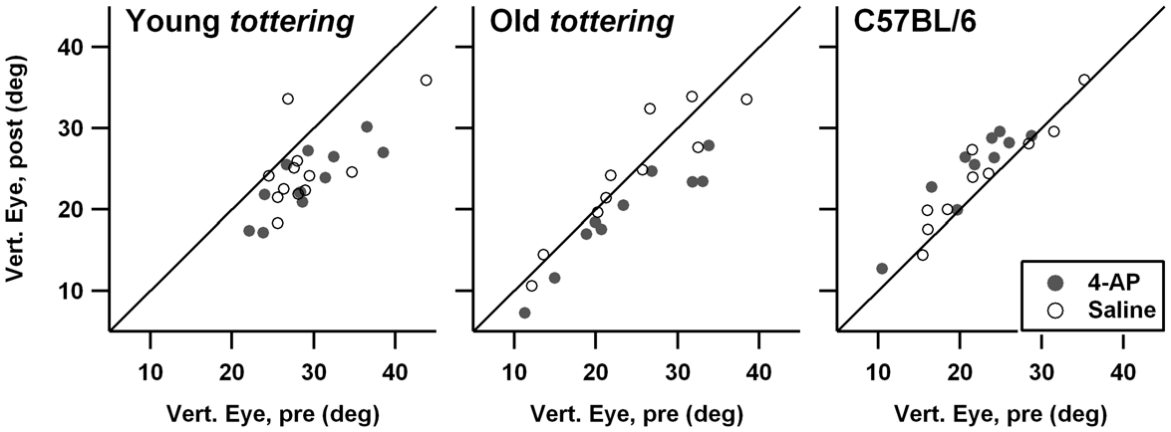
Comparison of resting vertical eye position before and after saline or 1.25 mg/kg 4-AP administration. Symbols and format are identical to Figure 1. Averaged drug and saline responses differed significantly in old *tottering*, but the difference may originate in an atypical response in the saline experiments.

Elevated resting eye positions have been demonstrated in *tottering* and speculated to be a homologue of downbeat nystagmus [48], a common eye movement abnormality seen in humans with cerebellar disorders, and one that has been demonstrated to respond to 4-AP [7]. Additionally, any increase of Purkinje cell firing rate or communication of mean firing rate to Purkinje cell targets (as has been postulated, see Introduction) would be expected to depress vertical eye position. Figure 9 compares resting vertical eye position in the light, before and after treatment. The young mutants replicate the mild elevation of the eyes seen in Cacna1a mutants in prior studies [39], [40], while the cohort of eight older animals did not, owing to the very low eye position (approximately 13°, averaged across sessions) of one mouse. Eye position tended to be lower in the post-treatment period in mutants and higher in controls (a phenomenon even more prominent in the low-dose 4-AP series, plots not shown). The average change in vertical eye position for saline and 4-AP differed significantly for old *tottering* (saline: −0.3±3.2°; 4-AP: −4.3±2.4°; p = 0.0053) but not for young *tottering* (saline: −4.1±4.4°; 4-AP: −5.7±2.9°; p = 0.31) or controls (saline: +1.1±2.5°; 4-AP: +2.7±2.5°; p = 0.17). Comparison of the old and young *tottering* plots raises the possibility that it was the failure of vertical eye position to fall post-saline, rather than an increased tendency of vertical eye position to fall post-drug, that is responsible for the apparent drug effect in old *tottering*. This possibility is re-enforced by the plots from the low-dose 4-AP experiments (not shown), in which the decline in eye position of old *tottering* post-saline was more typical, and the drug effect was non-significant (saline: −1.5±2.3°; 4-AP: −2.5±3.0°; p = 0.30).

### Intrafloccular 4-AP effects on rotation responses

We performed intrafloccular injections of 4-AP with two goals. First, they would provide insight into the extent that any behavioral effects of systemic administration reflect actions of the drug within the flocculus. Second, intracerebellar injections of aminopyridines have been claimed to normalize cerebellar cortex activity in ataxic mice [13], and the claim has been cited repeatedly in the clinical literature (for instance, see[7]). This speculation can be tested with intrafloccular injections of 4-AP, since considerable data are available regarding the relationships between firing patterns of floccular Purkinje cells and eye movements. Consequently, changes in floccular activity can be inferred from any changes in eye movements following drug administration. For the intrafloccular injection experiments, we studied only mutants, aged 3–8 months.

**Figure 10:**
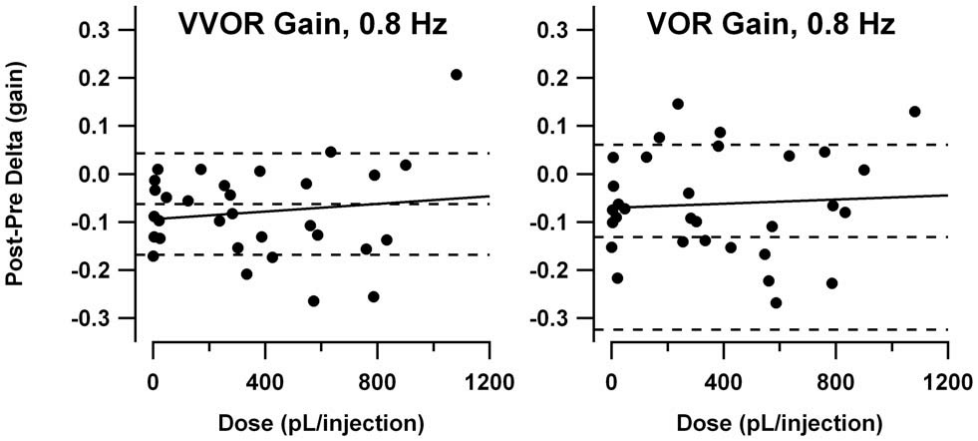
Change in VVOR gain (left panel) and VOR gain (right panel) following intrafloccular 4-AP injections in *tottering*, plotted as a function of volume of each injection. Horizontal dashed lines indicate the mean and ±2SD confidence interval for gain change in the systemic saline sessions of young *tottering*. 4-AP did not engender any changes in gain proportional to drug dosage.

Calculated ejection volumes ranged from 0.4 to 1081 pL/injection (median = 302 pL). Figure 10 plots changes in VVOR and VOR gain as functions of injection volume. Dashed horizontal lines indicate the mean and ±2SD confidence interval for gain change from the systemic saline injection experiments in young *tottering* (pooling experiments from both the 1.25 mg/kg and the earlier low-dose series). Since the systemic injection experiments were conducted in a fashion virtually identical to the intrafloccular injection experiments and used similarly aged mutants, they provide informative control data. The preponderance of datapoints lie at negative gain changes, indicating that, as in the systemic injection experiments, there was a tendency for gain to decline over the course of each experimental session, suggesting habituation. There was no systematic change in gain as a function of 4-AP dosage for either VOR or VVOR, and most data points fell within the gain variation observed in the systemic saline injection experiments. Thus intrafloccular injection of 4-AP had no effect on the response to head rotation in the light and darkness.

**Figure 11:**
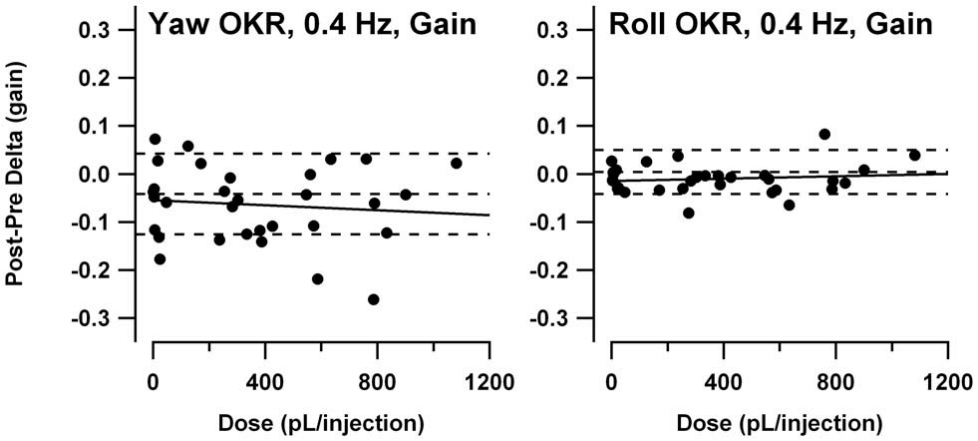
Change in sinusoidal OKR gain following intrafloccular 4-AP injections in *tottering*, plotted as a function of volume of each injection. Left panel: yaw OKR. Right panel: roll OKR. Horizontal dashed lines indicate the mean and ±2SD confidence interval for gain change in the systemic saline sessions of young *tottering*. 4-AP did not engender any changes in gain proportional to drug dosage.

Figure 11 shows the gain change vs. volume plots for 0.4 Hz sinusoidal yaw and roll OKR. For yaw rotations there was no systematic relationship between 4-AP dosage and gain changes. Most points fall at negative values of gain change, replicating the observation of habituation in the systemic injection experiments. For roll rotations there was again no gain-enhancing effect of 4-AP. In the systemic injection experiments there was no habituation effect. In the intrafloccular injection experiments, most of the gain changes fell below the mean for the saline experiments, suggesting a slight degree of habituation, but still one that is less than observed for yaw rotations. Thus the animals behaved very similarly in systemic and intrafloccular injection experiments, supporting the reproducibility of the observations on habituation, and the lack of any impact of 4-AP on sinusoidal OKR, by either route of administration.

**Figure 12:**
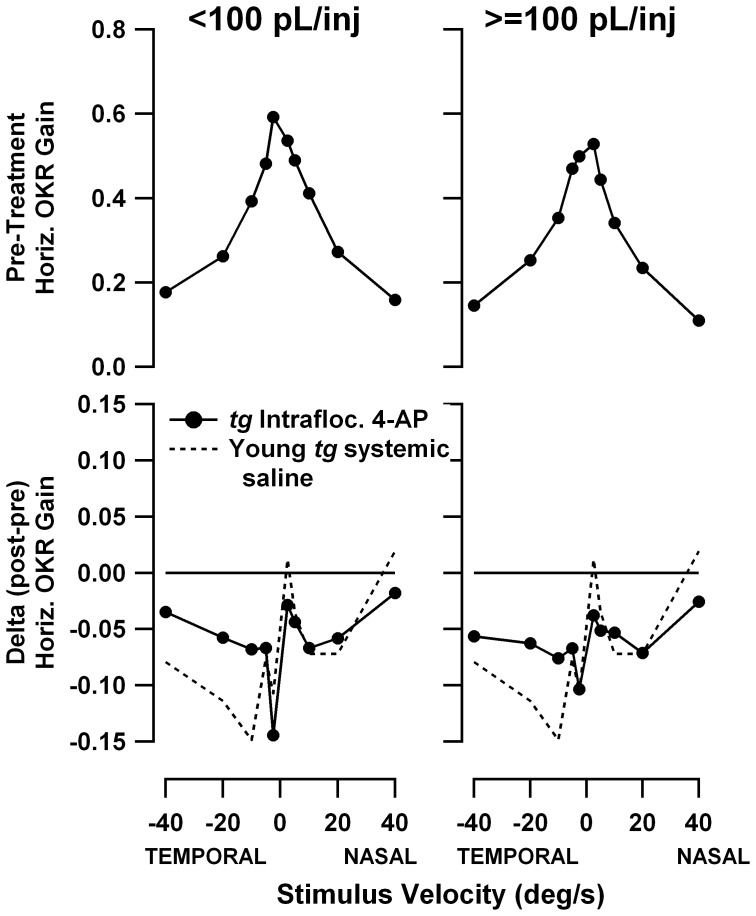
Effect of 4-AP on yaw OKR speed tuning, following low-volume (<100 pL/injection, left panels) and high-volume (>100 pL/injection, right panels) intrafloccular injections. Format of graphs is identical to Figures 6 and 7. In the gain change plots (bottom plots), the gain change curve for the systemic saline sessions of young *tottering* from Figure 6 has been superimposed for reference (dashed line). The gain change curves for the 4-AP intrafloccular injections and systemic saline do not differ, or do not differ according to the volume of injected 4-AP, suggesting the lack of any drug effect.

**Figure 13:**
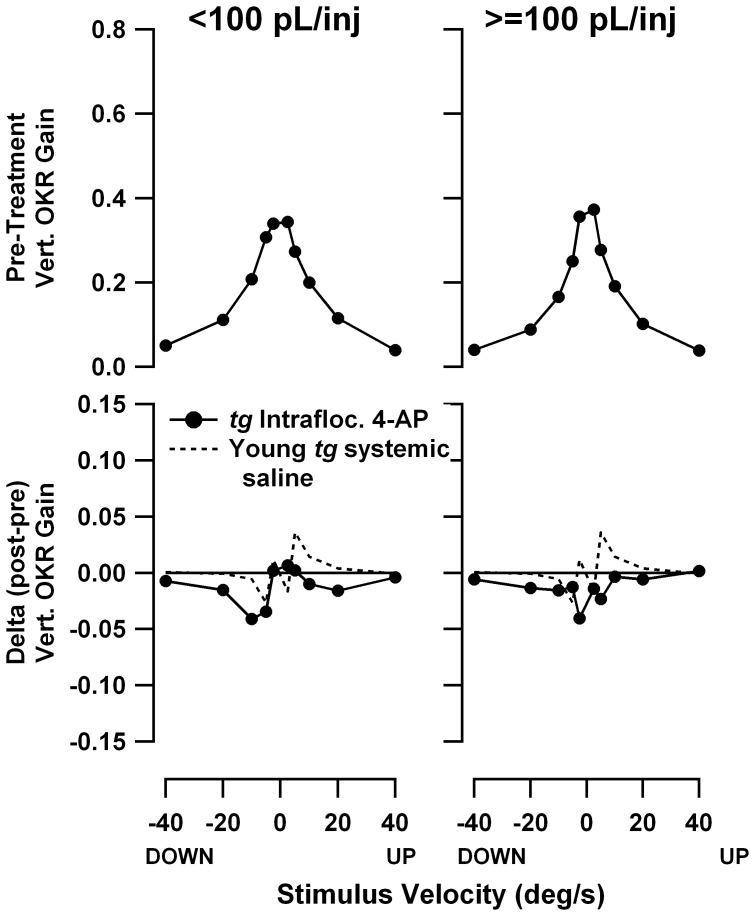
Effect of 4-AP on roll OKR speed tuning, following low-volume (<100 pL/injection, left panels) and high-volume (>100 pL/injection, right panels) intrafloccular injections. Format of graphs is identical to Figure 12, with dashed curves replicating the systemic saline curves for young *tottering* from Figure 7. There is little difference between the intrafloccular drug injection and systemic saline gain-change curves, nor any significant difference between low-volume and high-volume sessions, indicating the lack of any effect of 4-AP.

Figures 12 and 13 show pre-treatment speed tuning curves and treatment-associated gain changes for constant-velocity yaw and roll stimuli, respectively. Separate plots are shown for injection volumes below 100 pL/injection (left panels, median injection volume of 17pL) and above 100 pL/injection (right panels, median injection volume of 486 pL). The gain change plots also include the gain change curves from the systemic saline injections in young *tottering*, replicated from Figures 6 and 7 (dashed lines), which as noted above, provide informative control data. Yaw gains exhibited the mild decline familiar from the systemic administration experiments. There is no difference in the gain changes evoked by small and large 4-AP doses, an observation confirmed using plots of gain change vs. injection volume for responses to −10 and +10 °/s stimuli (plots not shown). Note that the V-shaped profiles of the yaw OKR gain changes following systemic 4-AP seen in Figure 6 were not replicated, indicating that if the differences in gain changes for saline and systemic 4-AP were real effects, they may be attributed to actions of 4-AP outside the cerebellar flocculus. Figure 13 demonstrates that intrafloccular 4-AP had no effect on the constant-velocity roll OKR. There is a tendency toward a decline in gains irrespective of injection volume but it is very weak, supporting other observations that roll OKR habituates somewhat less than yaw OKR.

**Figure 14:**
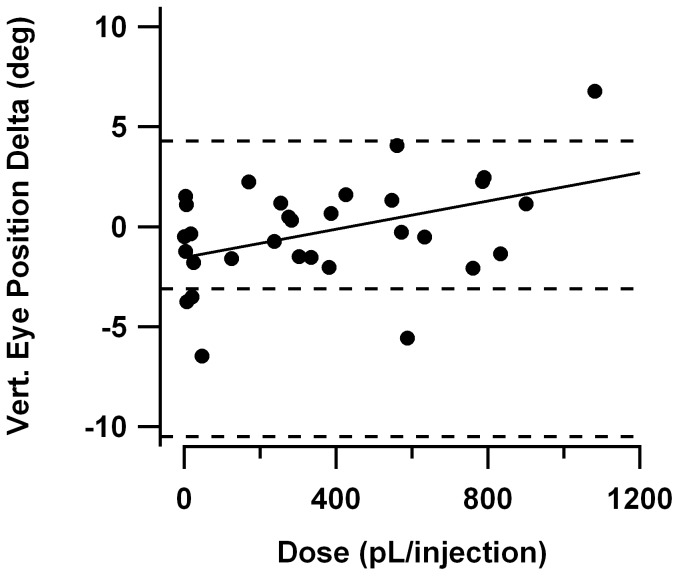
Change in vertical resting position of eye following intrafloccular 4-AP injections in *tottering*, plotted as a function of volume of each injection. Horizontal dashed lines indicate the mean and ±2SD confidence interval for change in resting position in the systemic saline sessions of young *tottering*. Regression curve has been superimposed. There was a weak but significant trend for eye elevation to increase as a function of 4-AP dosage, consistent with 4-AP reducing mean Purkinje cell firing rate.

Figure 14 plots the change in resting vertical eye position vs. injection volume, with dashed reference lines giving the mean and ±2SD confidence interval for eye position change from the systemic saline injection experiments in young *tottering*. The linear regression line is also superimposed. In the systemic saline experiments (as well as the systemic 4-AP sessions) there was a tendency toward lower eye positions in the post-treatment period. In contrast, following intrafloccular 4-AP there was a weak tendency for *less* depression of the eye in the post-treatment period (r^2^ = 0.19, p = 0.0007). Apart from the sessions in which injection volume was less than 100 pL, almost all the data points fall above the mean change for the saline sessions. This result is consistent with intrafloccular injection of 4-AP in the hundred micromolar range reducing, rather than increasing, the inhibitory tone exerted by the flocculus on its synaptic targets in the vestibular nucleus. This result is consistent with data in the slice preparation, in which there was a mild reduction of Purkinje cell firing rate following bath application of 4-AP above 5 µM [14].

## Discussion

We assessed the ability of 4-AP, administered systemically in *tottering* mice at doses above and below levels demonstrated to block *tottering's* dystonic episodes, to ameliorate ocular motor deficits that are attributable to hypofunction of the flocculus, a region of the vestibulocerebellum. Contradicting the prior claims (reviewed in Introduction) that aminopyridines rescue floccular function, we did not observe any consistent enhancement of flocculus-driven eye movements. There was no effect on *tottering*'s deficient amplitudes of VOR, VVOR, or sinusoidal OKR about the yaw and roll axes. An apparent effect on resting eye position that might be interpreted as a drug-induced enhancement of mean floccular Purkinje cell activity was seen in only the old *tottering* group and is arguably an artifact of a mildly atypical set of control (saline) data, rather than a true drug effect.

The only clear effect of systemic 4-AP we observed was a small enhancement of the OKR in response to constant-velocity, upward rotation of the visual surround with respect to the recorded eye, which occurred at both the 1.25 mg/kg dosage and in a separate series of experiments at which dosage was lower, averaging 0.7 mg/kg. This gain increase is unlikely to represent an enhancement of the flocculus response to optokinetic stimuli, for a number of reasons. First, since upward eye movements correspond to a decrease of simple spike firing rate in the ipsilateral flocculus and the vestibular nucleus receives direct projections from the ipsilateral flocculus only [61], [62], the gain increase cannot coincide with enhanced peak firing rates. Second, the lack of any improvement in the response to the upward hemicycle of sinusoidal optokinetic stimuli is inconsistent with an increased sensitivity of floccular Purkinje cells to optokinetic stimuli. Third, control animals also exhibited a gain enhancement, indicating that it does not arise as a reversal of the electrophysiological consequences of the Cacna1a mutation. Fourth, injection of 4-AP within the flocculus did not produce a similar gain enhancement. We excluded the possibility that the gain increases are an artifact of 4-AP engendering an upward drift of the eyes. Another possibility is that it arises from an effect on the fast phases of optokinetic nystagmus. Mice may fail to sustain OKR during unidirectional stimuli of even modest (a few seconds) durations [55], the eye slowing as it approaches a stable eccentric position. Conversely, the eye often moves more rapidly immediately following a fast phase of nystagmus, with an exponential deceleration suggesting that a post-saccadic glissade is being superimposed on the constant-velocity OKR. As discussed in Methods, we excluded from analysis portions of records where the eye was clearly drifting to an arrest or where it was moving more rapidly (but with an exponentially declining velocity) within the first tens of milliseconds following a fast phase. But we preferred to minimize such exclusions, so post-saccadic glissades as well as eccentric arrests may have influenced the speed tuning database slightly. As such, an increase in fast phases following 4-AP would bias the gain upward. Very little is known regarding the triggering mechanism for fast phases of vestibular and optokinetic nystagmus, but flocculectomy and cerebellectomy do not prevent the triggering of fast phases [45], [63], and the limited literature on fast phase generation has focused on extracerebellar loci [64], [65]. Thus if 4-AP enhanced the response to upward constant-velocity OKR via an effect on fast phase generation, the locus of that effect could well be extracerebellar, and if so, would not support claims that 4-AP restores flocculus function.

The other possible effect of systemic 4-AP, a reduction in the tendency for compensatory eye movement amplitudes to decline through the course of the recording session, was less consistent. This reduction–which we are loosely terming ‘habituation’ - tended to be present only in the mutant, but was restricted to certain behaviors, including the VVOR, VOR, and constant-velocity yaw OKR. Habituation was inconsistent during sinusoidal yaw OKR, being present in the high-dose and absent in the low-dose 4-AP series. Habituation was not observed (or at least, was much less prominent) for either sinusoidal or constant-velocity roll OKR. The normal flocculus in afoveate mammals modulates strongly during both horizontal and vertical OKR, and any generalized decline in flocculus modulation would be expected to affect the response about both axes. Thus the habituation may not arise in the flocculus and therefore, the mild effect of 4-AP may not originate there, either. The observation that 4-AP affected habituation in some behaviors but not others (specifically, sinusoidal yaw OKR), also argues against the mechanism of anti-habituation being a simple correction of the consequences of the Cacna1a mutation on flocculus electrophysiology. One possible explanation for the anti-habituation effect is that 4-AP produces a heightened state of alertness, enhancing vestibular reflexes in a manner comparable to amphetamines [66], and counteracting a decline in alertness specific to the mutants. We cannot exclude the possibility that the greater habituation for mutants masked a beneficial effect of 4-AP on *tottering's* eye movements. This possibility could be resolved by targeted experiments involving a single behavior (e.g., 0.8 Hz VOR), conducted rapidly enough to minimize habituation. However, the possibility seems small, since 4-AP also had no effect on the deficient gain of OKR about the roll axis, in which habituation was minimal.

Of note, the reason for lesser habituation to roll than to yaw stimuli is unclear. We cannot exclude the possibility that it relates to the sequence of the experiment, which proceeded in the following order: pre-treatment yaw, pre-treatment roll, treatment, post-treatment roll, post-treatment yaw. With the longer interval between pre-and post-treatment yaw stimuli than pre- and post-treatment roll stimuli, any gradual, generalized decline in gain through the experiment would have had a greater impact on the yaw gains. Additionally, there were more yaw stimuli of all kinds than roll stimuli in the battery, which might have caused disproportionate habituation in the yaw axis circuitry.

### Significance of current findings for human 4-AP literature

The bulk of the data on 4-AP's beneficial effects on eye movements of cerebellar patients has focused on its ameliorating downbeat nystagmus (DBN), an involuntary eye movement in which upward drifts of the eyes (the ‘slow phases’ of nystagmus) alternate with downward, saccadic, resetting fast phases [44]. DBN has been divided into three components, each of which is present to varying degrees in any specific patient. These include a gaze angle-dependent component attributed to dysfunction of the gaze-holding neural integrators, a gaze-independent component that is modulated by pitch angle of the head, and a gaze-independent, tilt-independent component [50], [67]. Although the origin of each component remains a matter of debate, flocculus hypofunction is argued to be a significant contributor. In support of this idea, metabolic imaging in DBN patients has revealed floccular hypometabolism [68], [69], the flocculus hypometabolism was lessened in association with an attenuation of DBN in a patient receiving 4-AP [68], DBN can be generated in monkeys by lesioning the flocculus [45], patients with DBN also exhibit deficiences of other eye movements known to be supported by the intact flocculus, including smooth pursuit and eccentric gaze holding [44], and some of these associated abnormalities also respond to 4-AP [7], [9], [11], [70].

Unfortunately, to date, classic spontaneous DBN has not been reported in ataxic mice (nor was it observed in the current experiments). Hyperactivity of the eye movement response to static pitch tilts (the tilt maculo-ocular reflex) has been suggested as a murine homolog of DBN, but that speculation has yet to be proven [48], [71]. Furthermore, whereas there are extensive data indicating a facilitating role of the flocculus in the eye movement responses to rotation, the role of the flocculus in the response to static tilts is uncertain, making it impossible to infer the changes in the outputs of vestibulocerebellar Purkinje cells on the basis of any drug-induced changes in the tilt maculo-ocular reflex. In sum, a direct investigation of the mechanism by which 4-AP ameliorates human DBN is still impossible in mice. Nevertheless, the current results in mice provide an important perspective on the human 4-AP literature. The finding that 4-AP, given at dosages near the maximum tolerated by mice, does not enhance flocculus function in *tottering* indicates the need to reassess the customary explanations for its beneficial effects in humans. If we accept the current consensus that DBN in humans is at least partially attributable to flocculus dysfunction, and the deficient rotational responses in *Cacna1a* mutants likewise arise from flocculus hypofunction [17], [40], then it becomes unreasonable to explain the effects of 4-AP in humans based on current knowledge of the effects on *tottering* Purkinje cells, obtained from regions outside the flocculus, studied *in vitro*. Either 4-AP in maximal tolerable dosages does not affect the flocculus of mice as it does in humans, or the benefits of 4-AP in humans do not arise from action at the flocculus, or 4-AP does not affect the flocculus as it does the regions of the mouse cerebellum in which its effects have been studied *in vitro* [14], or the Purkinje cell dysrhythmicity that 4-AP corrects in extrafloccular regions *in vitro* (which is also present in floccular Purkinje cells *in vivo*) is not the cause of the cerebellar dysfunction.

These implications are relevant specifically to ocular motor dysfunction attributable to hypofunction of the flocculus, and potentially to non-ocular motor abnormalities (e.g., trunk and limb ataxia) attributable to loss of cerebellar function. These reservations do not apply to the effects of 4-AP on the episodes of ataxia in humans with the CACNA1A channelopathy, EA-2. In that case, there is a clear homology between the human episodes of ataxia and murine episodes of dystonia [72], including the fact that 4-AP is effective in blocking the episodes in both humans and mice [12], [13]. Moreover, there is strong evidence that the episodic motor dysfunction in *tottering* depends on the integrity of cerebellar structure and presence of increased expression in the mutants of L-type voltage-activated calcium channels, and is also associated with increased cerebellar metabolism, and as such represents a gain-of-function disorder [72], [73]. The efficacy of 4-AP in blocking *tottering*'s episodes of dystonia contrasts with the lack of efficacy on its continuous deficient eye movements, and supports the likelihood that the two forms of motor dysfunction are etiologically distinct, and demonstrates that it is no longer appropriate to explain aminopyridine effects on human eye movements with reference to their effects on episodic dystonia in mice. Along the same lines, 4-AP has been demonstrated to reverse electrophysiological and behavioral defects in a murine model of spinocerebellar ataxia type 1, another instance of a gain-of-function mutation [42]. The current study does not apply to the conclusions of that study, except to underscore the problematic nature of explaining 4-AP actions in ataxic humans in terms of specific mouse models when the mechanisms of cerebellar dysfunction may differ.

### Limitations

One limitation of the current study is that it sampled only a limited set of eye movement abnormalities. Other abnormalities that have been demonstrated in *tottering* and likely reflect flocculus dysfunction were not assessed, including reduced vestibular plasticity, shortened time constants of gaze holding and the VOR, altered correspondence between the response and stimulus axes of the VOR, and higher gains of the response to static tilts [40], [48]. Nevertheless, the elements that we did assess are sufficient to demonstrate that 4AP does not ‘rescue’ the flocculus from the effects of the *tottering* Cacna1a mutation, whether by enhancing firing, enhancing modulation, or removing an irregularity that somehow nullifies normal activity.

A more significant set of limitations relates to 4-AP dosage. The dose we used for systemic administration was only just above the minimum dose that modulates *tottering's* dystonic attacks [13], opening the possibility that concentrations fell below the minimum effective levels. However, animals did not tolerate higher dosages. Moreover, intrafloccular administration of 4-AP in concentrations well above what would be achieved by systemic administration also failed to produce evidence of enhanced flocculus activity. Our non-effective intrafloccular injections do provide strong evidence that the effects of 4-AP on eye movements in humans cannot be explained by reference to the experiments in which dystonic events in *tottering* were successfully blocked by injections of 100 µM 3,4-diAP within the vermis. While our 4-AP concentration was higher, the efficacy of 4-AP when applied directly to neuromuscular junctions is approximately 6 times less than that of 3,4-diAP [74], so the degree of potassium channel blockade we engendered was likely comparable to that achieved in the vermis injection experiments.

It remains possible that we failed to demonstrate improvements in flocculus-supported eye movements analogous to those engendered in humans because the beneficial effects of 4-AP are restricted to a narrow range of tissue concentrations, one that we *exceeded* in our experiments. It is difficult to estimate the likelihood of this possibility because brain levels achieved following oral and subcutaneous 4-AP dosing, both in humans and mice, have not been reported. The 10 mg, oral dose of 4-AP used in human DBN treatment trials [8] has been reported to produce peak serum levels of approximately 50–120 ng/ml, with average mean levels of about 60 ng/ml in a single patient treated with 10 mg three times daily [26], [27], [75]. Accordingly, in the study demonstrating the ability of 4-AP administered in drinking water to improve rotarod performance in *tottering* [76], mice were dosed to generate a serum concentration of 50 ng/mL, but the actual levels generated in serum were not assessed. Likewise, the brain concentrations are unknown. In rats, the CSF:serum ratio for 4-AP at 30 minutes after intravenous administration is 0.21 [77]. Assuming a comparable brain penetrance in mice receiving continuous oral dosing, a 50 ng/mL serum concentration would predict brain levels of only 0.11 µM, considerably below the lowest tested concentration that affected Purkinje cell rhythmicity *in vitro* [14]. However, the CSF:serum ratio following chronic oral administration has not been reported, and is likely much higher than the ratio following a single intravenous dose. For comparison, our entire 1.25 mg/kg subcutaneous bolus, if absorbed and equilibrated throughout the body before significant elimination occurred, would engender a peak serum level of 1200 ng/mL (based on the reported volume of distribution of 1036 mL/kg in guinea pigs [78]). Again applying the CSF:serum ratio from rats, this serum level would predict a peak CSF concentration of 2.7 µM, which does fall within the range demonstrated to affect Purkinje cell rhythmicity *in vitro*. More data are required to determine if brain levels in the current experiments in fact exceeded those that were effective on rotarod performance. However, the likelihood that we missed an effect by overshooting a narrow therapeutic window is rendered less likely by our intrafloccular injection experiments, which do not suggest that efficacy declines as dose increases beyond some range (see Figures 10, 11, and 14).

### Concluding comments; future directions

The current results demonstrate the uncertainties in the mechanism by which 4-AP improves eye movement deficits in ataxic humans, and highlight uncertainties in the origin of ataxia in *tottering*, a murine model of cerebellar dysfunction that has provided much of the basis for claims as to those mechanisms of action. One potentially informative line of inquiry would be to determine the brain levels of 4-AP that were achieved by oral dosages that were demonstrated to improve rotarod performance [14], and to test the effects of those levels *in vitro* on Purkinje cells from vestibulocerebellum. A second approach would be to record floccular Purkinje cells *in vivo* under the influence of aminopyridines, administered by routes and at dosages that produce improvements in the continuous forms of motor function (as opposed to blocking the episodic dystonia). If floccular Purkinje cells increase their regularity as predicted by the *in vitro* experiments outside the flocculus [14] and eye movements again fail to improve, then the hypotheses that cerebellar dysfunction in *tottering* relates to Purkinje cell irregularity, and that 4-AP improves motor function in *tottering* (and by implication, in other cerebellar disorders) by reducing irregularity [14] are suspect, and attempts to find alternatives to 4-AP by screening drugs for their ability to regularize Purkinje cell firing will likely fail. Finally, further inquiries into the origin of the improvement in upward-directed OKR are warranted. Our data suggest that the site of action may lie outside the flocculus, and possibly outside the cerebellum. Identifying the site of action may reveal a hitherto unsuspected mechanism by which aminopyridines affect eye movements.
